# Visual multiple cross displacement amplification for the rapid identification of *S. agalactiae* immediately from vaginal and rectal swabs

**DOI:** 10.1186/s13568-020-01168-3

**Published:** 2021-01-06

**Authors:** Xueqin Cheng, Zhiqian Dou, Jing Yang, Dexi Liu, Yulong Gu, Fenglin Cai, Xiaobing Li, Meifang Wang, Yijun Tang

**Affiliations:** 1 Department of Respiratory and Critical Medical, Taihe Hospital, Hubei University of Medicine, Shiyan, Hubei China; 2 Hubei Key Laboratory of Embryonic Stem Cell Research, Tai-He Hospital, Hubei University of Medicine, Shiyan, China; 3 Department of Gynaecology and Obstetrics, Taihe Hospital, Hubei University of Medicine, Shiyan, Hubei China; 4 Department of Pharmacy, Wuhan General Hospital of the Chinese People’s Liberation Army, Wuhan, Hubei China; 5 Department of Stomatology, Taihe Hospital, Hubei University of Medicine, Shiyan, Hubei China; 6 Department of Clinical Laboratory, Taihe Hospital, Hubei University of Medicine, Shiyan, Hubei China

**Keywords:** *Streptococcus agalactiae*, Multiple cross displacement amplification, Detection limit

## Abstract

*Streptococcus agalactiae* (*S. agalactiae*) is an important pathogen that can lead to neonatus and mother infection. The current existing techniques for the identification of *S. agalactiae* are limited by accuracy, speed and high-cost*.* Therefore, a new multiple cross displacement amplification (MCDA) assay was developed for test of the target pathogen immediately from vaginal and rectal swabs. MCDA primers screening were conducted targeting *S. agalactiae pcsB* gene, and one set of MCDA primers with better rapidity and efficiency was selected for establishing the *S. agalactiae-*MCDA assay. As a result, the MCDA method could be completed at a constant temperature of 61 °C, without the requirement of special equipment. The detection limit is 250 fg (31.5 copies) per reaction, all *S. agalactiae* strains displayed positive results, but not for non-*S. agalactiae* strains. The visual MCDA assay detected 16 positive samples from 200 clinical specimen, which were also detected positive by enrichment/qPCR. While the CHROMagar culture detected 6 positive samples. Thus, the MCDA assay is prefer to enrichment/qPCR and culture for detecting *S. agalactiae* from clinical specimen. Particularly, the whole test of MCDA takes about 63.1 min, including sample collection (3 min), DNA preparation (15 min), MCDA reaction (45 min) and result reporting (6 s). In addition, the cost was very economic, with only US$ 4.9. These results indicated that our *S. agalaciae*-MCDA assay is a rapid, sensitive and cost-efficient technique for target pathogen detection, and is more suitable than conventional assays for an urgent detection, especially for 'on-site' laboratories and resource-constrained settings.

## Introduction

*Streptococcus agalactiae* is a gram-positive group B Streptococcus (GBS) that is associated with the asymptomatic colonization of human urogenital and gastrointestinal tracts (Kwatra et al. 2016; Vieira et al. 2019). However, this opportunistic pathogen can cause serious infections in susceptible hosts, especially in neonates, leading to pneumonia, sepsis and meningitis (Boyer et al. 1985; McGee et al. 2010; Rosa-Fraile et al. 2017). As *S. agalactiae* could be vertically transmitted from a colonized mother to her babies, prenatal screening was recommended for those gestational age from 35 to 37 weeks or those who in faced with the risk of premature labor in industrial countries (Verani et al. 2010; Rabaan et al. 2017). When the positive results were reported, those high-risk pregnant women will accept antibiotic treatment to prevent the occurrence of vertical transmission (Curry et al. 2018). Owing to these prevention strategies, the incidence of neonatal diseases attributing to *S. agalactiae* significantly reduced in western countries (Verani et al. 2010). However, pregnant women who screened positive and received antibiotic treatment whose babies subsequently still infected with *S. agalactiae.* This may be due to the fact that *S. agalactiae* colonization was intermittent (Hansen et al. 2004; Feuerschuette et al. 2018). When antenatal screening conducted more than a certain time before delivery could not effectively predict the risk of infection (Yancey et al. 1996). Therefore, rapid screening techniques at the time of delivery were needed to do to prevent the occurrence of serious early-onset disease.

Currently, the gold standard used for the detection and identification of *S. agalactiae* was the enrichment culture, followed by biochemical analyses (Verani et al. 2010; Tickler et al. 2019). As the enrichment culture usually takes 48–72 h for obtaining the final result, which is a too long time for those women at delivery (Venkatesh et al. 2010; Connell et al. 2007; Kim et al. 2015). Moreover, the enrichment culture is with low sensitivity, leading to some false-negative results (Lin et al. 2019). Thus, a more sensitive method is required to complement the current method. Then, many rapid and sensitive molecular methods have been developed for *S. agalactiae*, and 4 commercially available GBS molecular diagnostic tests based on qPCR were applied in detecting *S. agalactiae* from Lim Broth culture about 18–24 h of vaginal-rectal swab specimens, including the Panther Fusion GBS assay, the BD MAXTM GBS assay, the ARIESGBS assay and the Xpert GBS LB assay (Shin et al. 2019; Vieira et al. 2019). Although these qPCR reduced the turn-around time and increased the sensitivity, it depends on a specialised laboratory and well-trained personnel, which limited its apply in primary-level medical and health care institutions. The other loop-mediated isothermal DNA amplification techniques were also developed for detecting *S. agalactiae* in clinical specimens, and the results were usually evaluated by the real time turbidimeter (a GENIE II fluorometer). However, the cost of this device was about £ 5000, which is still a burden in resource-limited hospitals in developing countries. Thus, a more rapid, free of special equipment and cost test of *S. agalactiae* directly from patient specimens is in an urgent need to satisfy the clinical requirement.

A new multiple cross displacement amplification technique was developed in recent years (Wang et al. 2017). This method avoid the long turn-around-time that required for enrichment culture, and the complex devices which is required for qPCR. It could be completed at a constant temperature in 45 min. A set of ten primers were designed in the MCDA assay, including displacement primers F1 and F2, cross primers CP1 and CP2, and amplification primers C1, C2, D1, D2, R1 and R2. These primers anneal to the target gene, and the polymerase extends in tandem yielding different-sized products, including CP1/D1 products, CP1/C1 products, R1/R1s products, and so on (Wang et al. 2017). Compared to the six primers of the LAMP assay, the MCDA assay of ten primers in theory is more specific. Moreover, according to the published articles, the LAMP assay is not as sensitive as the MCDA assay (Zhao et al. 2019a, b). Here, the judgment of the result was obtained by naked eyes without opening the lid, and thus preventing the aerosol pollution. All these merits made it a promise tool for point-of-care test for pathogen.

In this study, we developed a visual MCDA assay for *S. agalactiae* targeting *pcsB* gene, which can achieve rapid, sensitive and specific detection of the target pathogen. To further confirm the clinical application value of the MCDA assay, we collected 200 vaginal and rectal swabs from pregnant women, and compared it to the detecting result of the enrichment/qPCR assay and culture.

## Materials and methods

### Reagents

We purchased TIANamp Bacteria DNA Kits from Tiangen Biotech Co., Ltd.(Beijing, China). Lysozyme and agarose were purchased from Beyotime Biotechnology Co., Ltd.(Shanghai, China). The isothermal amplification kits and Malachite Green were purchased from Huidexin Bio-technology Co., Ltd.(Tianjin, China). qPCR Mix, pMD19-T Vector and DH5α were purchased from Takara Biomedical Technology Co., Ltd. (Beijing, China). The The swabs were purchased from Copan Diagnostics, Inc. (Lombardy, Italy). Todd Hewitt medium, gentamicin and nalidixic were obtained from Qingdao Hi-Tech Industrial Park Haibo Biotechnology Co., Ltd(Qingdao, China).

### Genomic DNA extraction

The analytical specificity of the MCDA assay was evaluated by 28 strains (Table 1), including 7 *S. agalactiae* strains and 21 non-*S. agalactiae* strains. *S. agalactiae* strain ATCC12386 was employed as a standard strain in this study. We collected these strains from the Department of microbial Laboratory, Taihe Hospital of Hubei University of Medicine, and identified these strains by DL-96 systems using 96E ID Card and conventional PCR targeting 16s rDNA and sequencing. Genomic DNA were extracted using a DNA Mini Kit following the manufacturers instructions, and extracted DNA was stored at – 80 °C until use. The purity and concentration of these extracted genomic DNA was determined using ultraviolet spectrophotometer (Nano drop one, Thermo, Massachusetts, USA) at A260/280.

**Table 1:** Strains used in this study and the results of MCDA assays

Bacteria	Strain no./source	No. of strains	MCDA-LFB result^2^
*Streptococcus agalactiae*	ATCC12386	1	+
	Isolated strains	6	+
*Streptococcus pyogenes*	ATCC19615	1	−
*Streptococcus pneumoniae*	ATCC6305	1	−
*Streptococcus mitis*	Isolated strain	1	−
*Streptococcus salivarius*	Isolated strain	1	−
*Streptococcus sanguinis*	Isolated strain	1	−
*Streptococcus dysgalactiae*	Isolated strain	1	−
*Streptococcus gordonii*	Isolated strain	1	−
*Streptococcus sinensis*	Isolated strain	1	−
*Streptococcus constellatus*	Isolated strain	1	−
*Streptococcus anginosus*	Isolated strain	1	−
*Enterococcus faecium*	Isolated strain	1	−
*Enterococcus raffinosus*	Isolated strain	1	−
*Staphylococcus epidermidis*	Isolated strain	1	−
*Staphylococcus saprophyticus*	Isolated strain	1	−
*Lactobacillus jensenii*	Isolated strain	1	−
*Micrococcus yunnanensis*	Isolated strain	1	−
*Pseudomonas aeruginosa*	Isolated strain	1	−
*klebsiella pneumoniae*	ATCC700603	1	−
*Escherichia coli*	Isolated strain	1	−
*Candida albicans*	Isolated strain	1	−
*Candida tropicalis*	Isolated strain	1	−

^1^+, positive; −, negative

^2^THH, Taihe Hospital; Sa, *Streptococcus agalactiae*

### Primer design

**Fig. 1:**
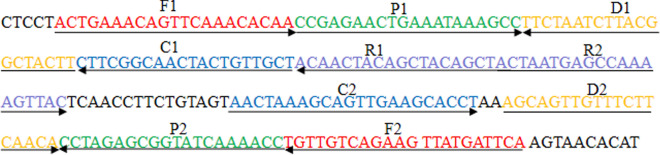
MCDA primer design for *pcsB* gene

The *pcsB* gene encodes a putative peptidoglycan hydrolase, which is necessary for cell wall. Here, the software PRIMER Premier 5.0 and PrimerExplorer V4 (http://primerexplorer.jp/elamp4.0.0/index.html) were used to design MCDA primers targeting *pcsB* gene of *S. agalactiae*. The MCDA primers were demonstrated in Table 2 and Additional file 1: Table S1, and the schematic diagram was showed in Fig. 1. All MCDA primers were synthesized and purified by TsingKe Biotech Co., Ltd. (Beijing, China) at HPLC purification grade.

**Table 2:** The set of MCDA primers used in this study

Primers name	Sequences	Length	Gene
pcsB-2F1	5′-ACTGAAACAGTTCAAACACAA-3′	21 nt	pcsB
pcsB-2F2	5′-TGAATCATAACTTCTGACAACA-3′	22 nt	
pcsB-2CP1	5′-AGCAACAGTAGTTGCCGAAGCCGAGAACTGAAATAAAGCC-3′	40 mer	
pcsB-2CP2	5′-AACTAAAGCAGTTGAAGCACCTGGTTTTGATACCGCTCTAGG-3′	42 mer	
pcsB-2C1	5′-AGCAACAGTAGTTGCCGAAG-3′	20 nt	
pcsB-2C2	5′-AACTAAAGCAGTTGAAGCACCT-3′	22 nt	
pcsB-2D1	5′-AAGTAGCCGTAAGATTAGAA-3′	20 nt	
pcsB-2D2	5′-AGCAGTTGTTTCTTCAACA-3′	19 nt	
pcsB-2R1	5′-AGCTGTAGCTGTAGTTGT-3′	18 nt	
pcsB-2R2	5′-ACTAATGAGCCAAAAGTTAC-3′	20 nt	

*nt* nucleotide, *mer* monomeric

### MCDA reactions

The *pcsB*-MCDA reaction reagents were prepared as follows: 0.4 µM displacement primers F1 and F2, 0.8 µM amplification primers C1, C2, D1, D2, R1 and R2, 1.6 µM cross primer CP1 and CP2, 1 µL Bst DNA polymerase (10U), 12.5 µL 2*Reaction Buffer, 1.5 µL Malachite Green and 1 µL DNA template.

The *pcsB*-MCDA products were analyzed by Malachite Green (MG), and further confirmed by gel electrophoresis. MG caused the color of reaction solution turning from green to bright green at the end of amplification for *S. agalactiae*, but turning colorless for non-*S. agalactiae*. A ladder-like band appeared at 2% gel electrophoresis for *S. agalactiae*, but no ladder-like bands were observed for non-*S. agalactiae.* Six different temperatures (ranging from 60 °C to 65 °C at 1 °C interval) were selected to evaluate the optimum amplification temperature.

### Sensitivity and specificity of the MCDA assay

Under the optimum condition, a tenfold serial dilution (from 2.5 ng to 2.5 fg) of *S. agalactiae* templates (ATCC12386) or the pMD19-T plasmid (from 3.15 * 10^6^ copies to 3.15 copies) was prepared for testing the sensitivity of the MCDA assay. The pMD19-T plasmid contained the target gene of *pcs B*, which was constructed according to the vector construction protocol. The specificity of the *S. agalactiae*-MCDA assay was evaluated using genomic DNA extracted from *S. agalactiae* and non-*S. agalactiae* strains.

### *S. agalactiae *culture

The vaginal and rectal swabs were inoculated on CHROMagar plates, and incubated at 37 °C for 24 h. The suspicious purple colonies were identified by the CAMP test, and further determined by conventional PCR and sequencing. The primer sequences were 8F(5′-AGAGTTTGATCCTGGCTCAG-3′) and 1492R(5′-GGTTACCTTGTTACGACTT-3′) (Turner et al. 1999).

### *S. agalactiae*-MCDA analysis of vaginal and rectal swabs

In order to further confirm the practicability of the *S. agalactiae*-MCDA assay, we directly collected 200 vaginal and rectal swabs from pregnant women with gestational age ≥ 24 weeks. Those pregnant women who used antibiotics in the 30 days prior to specimen collection were excluded. The swabs were transported at Copan’s medium. Each swab head circled about 10 times in 80 µL DW, and 20 µL were used for CHROMagar culture. We also added the other 20 µL to Todd Hewitt selective medium for enrichment culture about 24 h, and the DNA was extracted as ever reported (Vieira et al. 2019). The remaining solution were boiled at 100 °C for 15 min. When the temperature of the solution declined to room temperature, 2 µL DNA were directly used for MCDA detection**.** The qPCR primer sequences were 5′-TTTCACCAGCTGTATTAGAAGTA-3′ and 5′-GTTCCCTGAACATTATCTTTGAT-3′ (Vieira et al. 2019). We conducted qPCR reactions in a 20 µL volume that contained 0.4 µM forward and reverse primer each, 10 µL TB Green Fast qPCR Mix, and 2.0 µL DNA. The amplification was conducted at 95 °C for 30 s, followed by 40 cycles of 95 °C for 5 s, 60 °C for 34 s.

## Results

### Primers screening

**Fig. 2:**
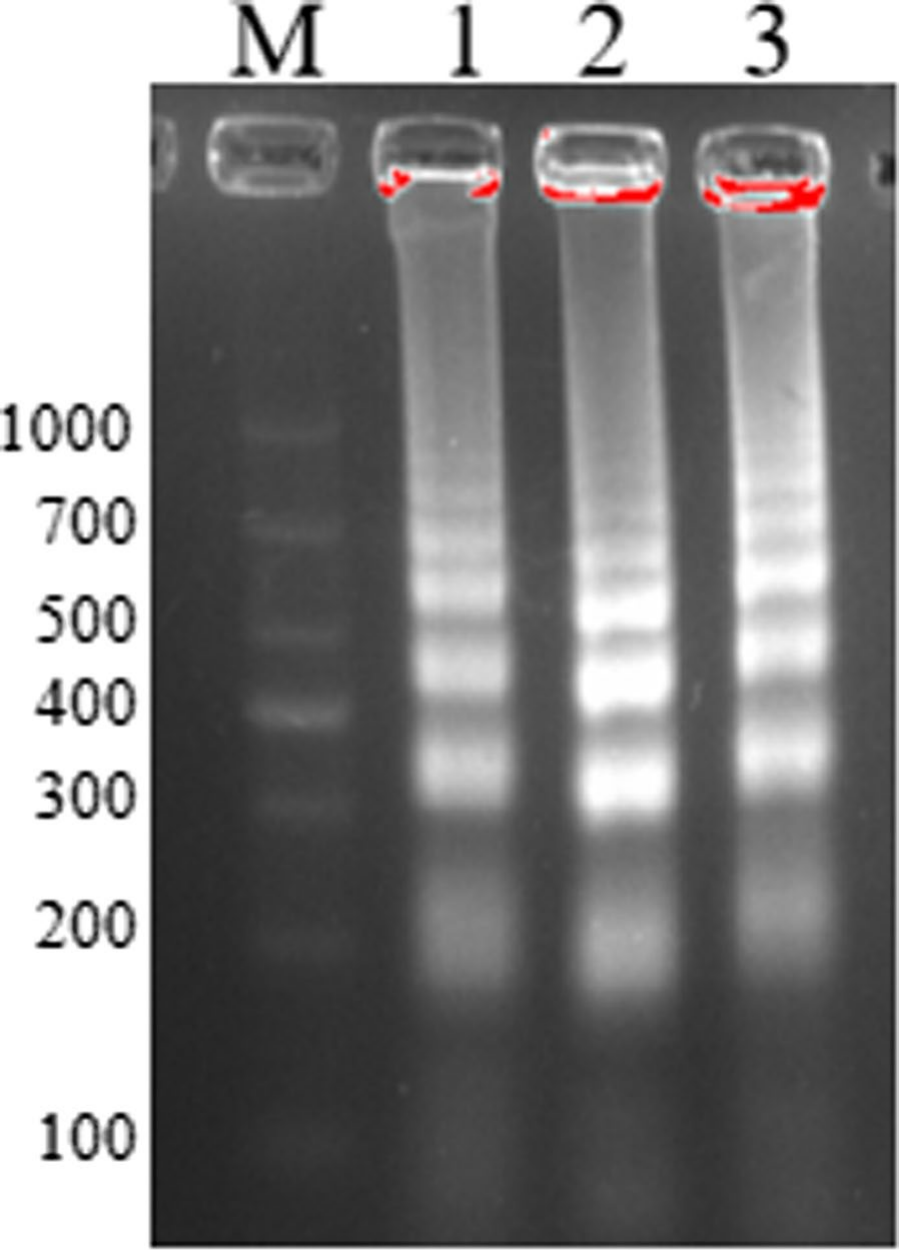
MCDA primers screening. The amplification performance of three sets of MCDA primers were evaluated by agarose gel electrophoresis. Lane M, DNA maker DL1000. Lane1/2/3, the first pcsB-MCDA primer set, the second pcsB-MCDA primer set, the third pcsB-MCDA primer set

In order to achieve the optimum amplification situation of *S. agalactiae*, three sets of MCDA primers targeting *pcsB* gene were designed. All these primers were conducted in MCDA reaction. 2% agarose gel electrophoresis was used to analyze the MCDA products of these three sets of MCDA primers. As observed in Fig. 2, the results showed that the ladder bands for the second set of MCDA primers were the brightest. Thus, the second set of MCDA primers was employed for the following experiments.

### Confirmation of MCDA products

**Fig. 3:**
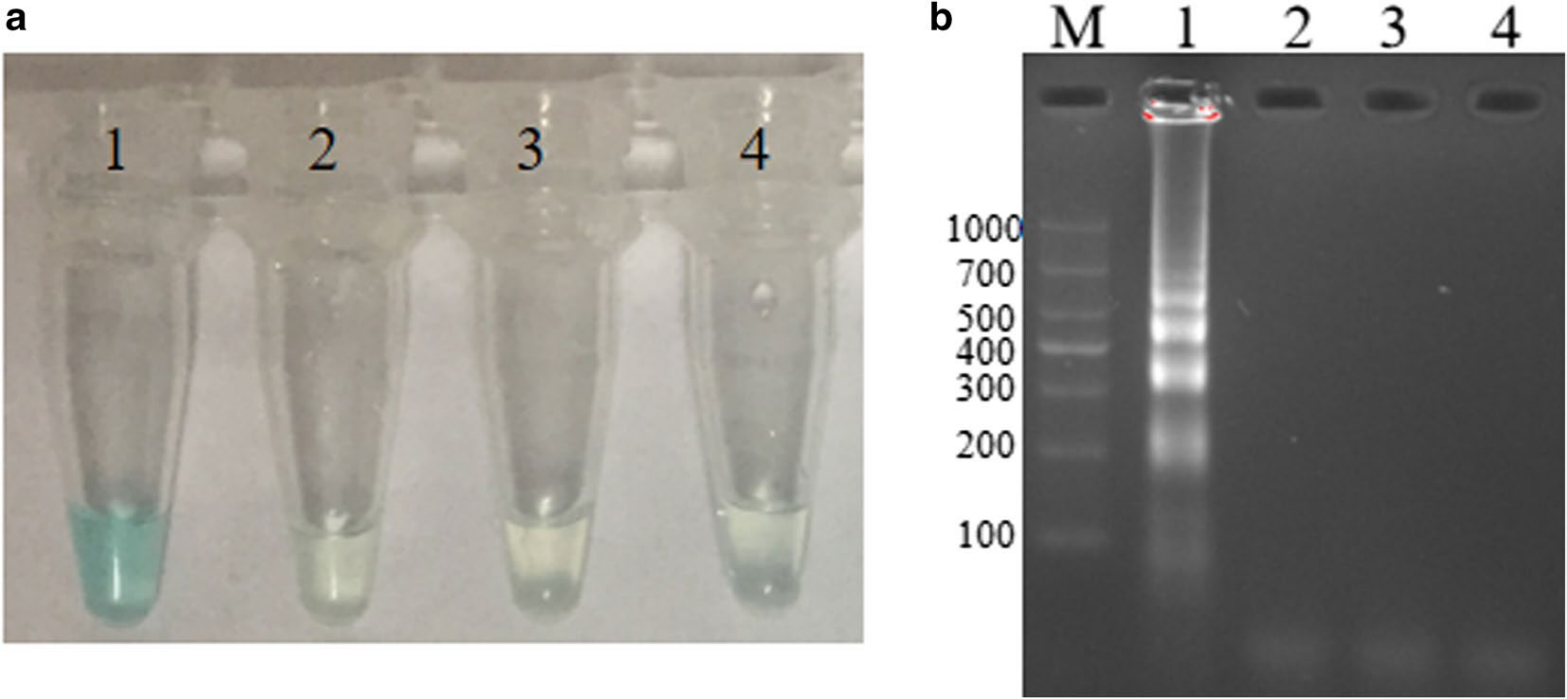
Confirmation of *S. agalactiae*-MCDA products. Methods of Malachite Green (**a**) and 2% Agarose gel electrophoresis (**b**) were used to analyze MCDA products. Lane M, DNA maker DL1000. Tube1/Lane1, positive group (*S. agalactiae* strain ATCC12386); Tube2/Lane2, negative group (*Streptococcus pyogenes*); Tube3/Lane3, negative group (*S. pneumoniae*); Tube4/Lane4, blank group (DW)

Three groups of genomic DNA were prepared to confirm the feasibility of the secreeded MCDA primers, including the first group of *S. agalactiae* ATCC12386, the second group of *S. pyogenes*, and the third group of *S. pneumoniae*. These DNA were respectively added to the MCDA mixture, and incubated at 62 °C for 1 h. At the end of amplification, MG and gel electrophoresis were employed to analyze MCDA products. By MG, light green were visual for *S. agalactiae*, and colorless were visual for *S. pyogenes*, *S. pneumoniae* and DW (Fig. 3). A ladder-like bands appeared at 2% gel electrophoresis for *S. agalactiae*, but no ladder-like bands were observed for negative control of *S. pyogenes*, *S. pneumoniae* and blank control of DW (Fig. 3). These results demonstrated that the second set of MCDA primers designed here could be applied in detecting *S. agalactiae*.

### The optimum amplification temperature of the* S. agalactiae*-MCDA assay

**Fig. 4:**
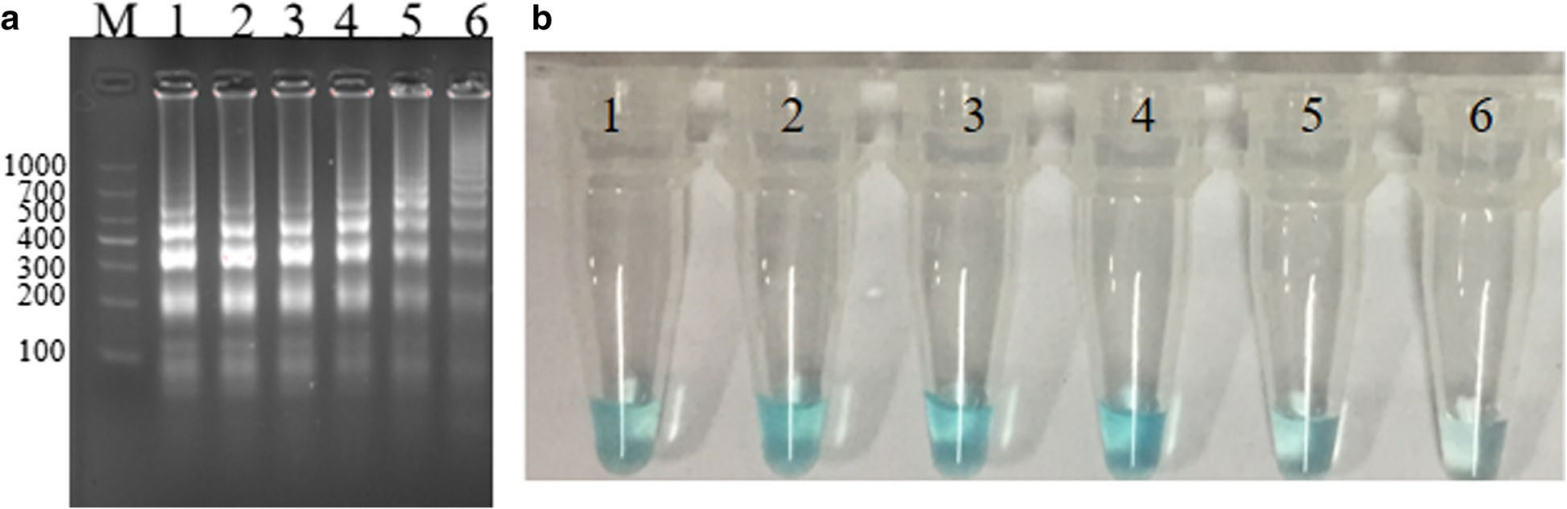
Temperature screening. 2.5 pg *S. agalactiae* strain ATCC12386 genome in MCDA reaction were respectively amplified at six different temperatures varing from 60 to 65 °C with 1 °C intervals. Then, we evaluate the amplification efficiency of the *S. agalactiae-*MCDA assay by using two methods, including Malachite Green and Agarose gel electrophoresis

The optimum temperature screening of the *S. agalactiae*-MCDA reaction were conducted at six different temperatures varing from 60 to 65 °C with 1 °C intervals for 45 min. As observed in Fig. 4, the color of tube 1, tube 2, tube 3 and tube 4 were almost the same, and were more darker than that of tube 5 and tube 6. By 2% agarose gel electrophoresis, the brightest ladder bands were observed at 61 °C. Thus, the subsequent experiments were completed at 61 °C.

### Sensitivity of the* S. agalactiae*-MCDA assay

**Fig. 5:**
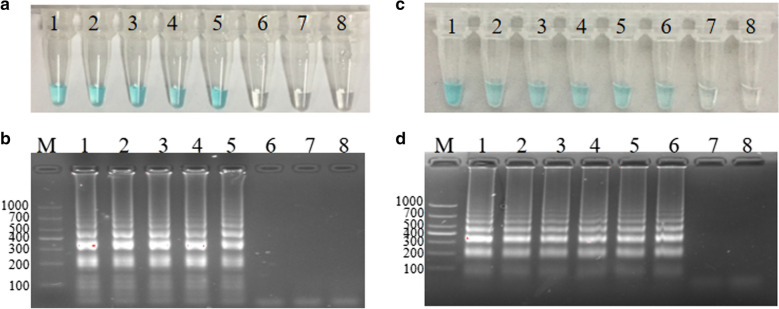
Sensitivity analysis. Tenfold serial dilutions of the template (2.5 ng, 250 pg, 25 pg, 2.5 pg, 250 fg, 25 fg, 2.5 fg) or the pMD19-T plasmid(3.15 * 10^6^ copies, 3.15 * 10^5^ copies, 3.15 * 10^4^ copies, 3.15 * 10^3^ copies, 3.15 * 10^2^ copies, 3.15 * 10 copies, 3.15 copies) were added to standard MCDA reactions and incubated at 61 °C for 1 h, respectively. Diagnosis techniques, including colorimetric indicator (**a**, **c**) and gel electrophoresis (b,d) were used for analysis of MCDA amplicons. Tubes (**a**)/Lanes (**b**) 1–8 respectively represent *S. agalactiae* strain ATCC12386 DNA levels of 2.5 ng, 250 pg, 25 pg, 2.5 pg, 250 fg, 25 fg, 2.5 fg per reaction, and a blank control (DW). Tubes (**c**)/Lanes (**d**) 1–8 respectively represent the pMD19-T plasmid levels of 3.15 * 10^6^ copies, 3.15 * 10^5^ copies, 3.15 * 10^4^ copies, 3.15 * 10^3^ copies, 3.15 * 10^2^ copies, 3.15 * 10 copies, 3.15 copies per reaction, and a blank control (DW)

Sensitivity of the MCDA assay was analyzed using *S. agalactiae* ATCC12386 genome and the pMD19-T plasmid. The genome were diluted from 2.5 ng µL^−1^ to 2.5 fg µL^−1^. The plasmid were diluted from 3.15 * 10^6^ copies µL^−1^ to 3.15 copies µL^−1^. Then, the different concentration of DNA was added to the MCDA reaction, which was incubated at 61 °C for 1 h. As observed in Fig. 5, the results showed that the detection limit of the MCDA assay was 250 fg µL^−1^ (31.5 copies µL^−1^), which was in accordance with that of the gel electrophoresis detection.

### Specificity of the* S. agalactiae*-MCDA assay

**Fig. 6:**
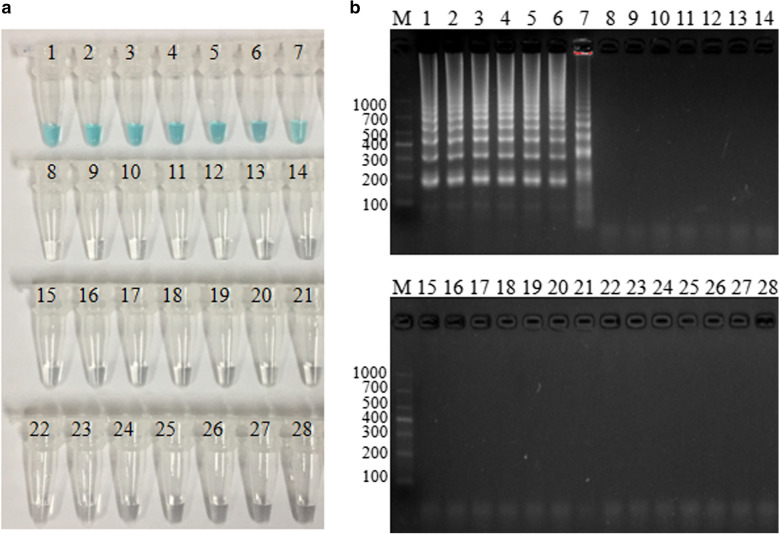
Specificity analysis. A total of 28 strains were used to evaluate the specificity of MCDA-LFB assays. Light green was visuable for all S*treptococcus agalactiae*, and colorless was observed for non*-Streptococcus agalactiae*. 1, Positive control (S*treptococcus agalactiae* strain ATCC12386), 2–7, *Streptococcus agalactiae*. 8, *Streptococcus pyogenes*; 9, *Streptococcus pneumoniae*; 10, *Streptococcus mitis*; 11, *Streptococcus salivarius*; 12, *Streptococcus sanguinis*; 13, *Streptococcus dysgalactiae*; 14, *Streptococcus gordonii*; 15, *Streptococcus sinensis*; 16, *Streptococcus constellatus*; 17, *Streptococcus anginosus*; 18, *Enterococcus faecium*; 19, *Enterococcus raffinosus*; 20, *Staphylococcus epidermidis*; 21, *Staphylococcus saprophyticus*; 22, *Lactobacillus jensenii*; 23, *Micrococcus yunnanensis*; 24, *Pseudomonas aeruginosa*; 25, *klebsiella pneumoniae*; 26, *Escherichia coli*; 27, *Candida albicans*; 28, *Candida tropicalis*

DNA extracted from 7 *S. agalactiae* strains and 21 non-*S. agalactiae* strains were used to evaluate the specificity performance of the MCDA assay. As observed in Fig. 6, all these *S. agalactiae* strains showed positive results, where light green was visual. However, colourless was visual for non-*S. agalactiae* strains.

### Using the MCDA assay with vaginal and rectal swabs

**Fig. 7:**
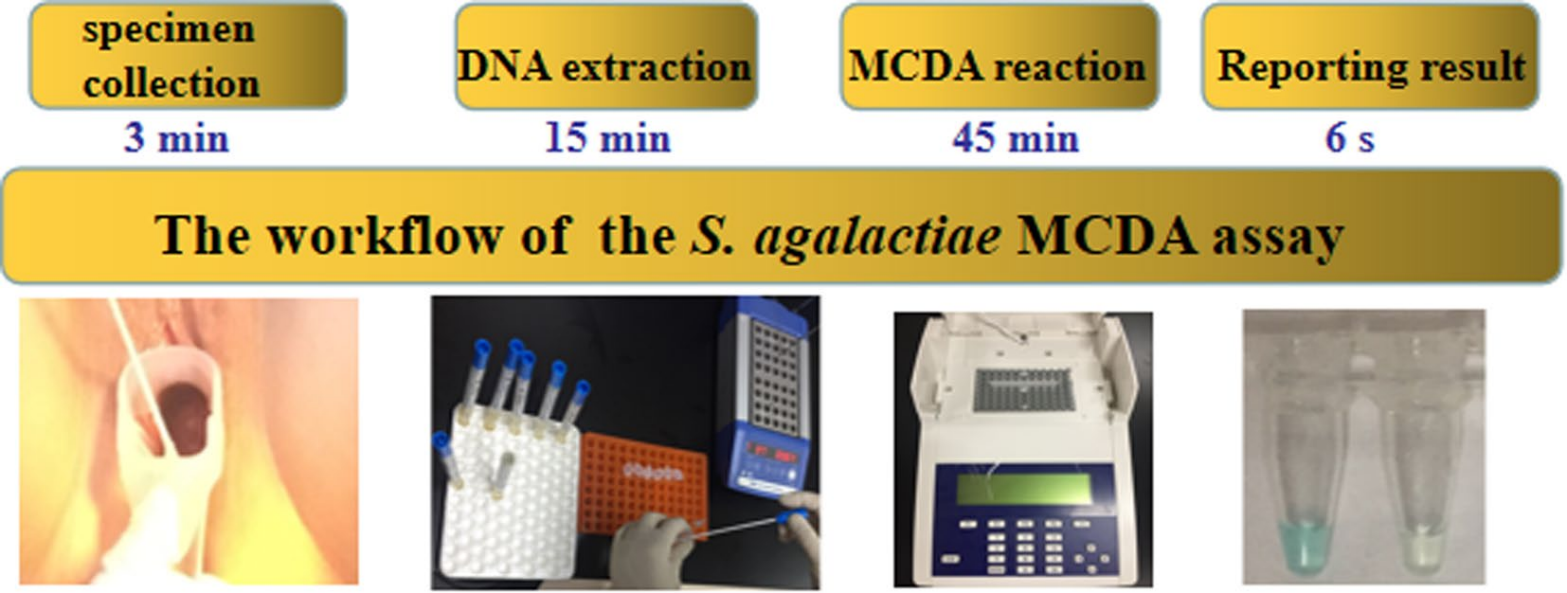
The workflow of the *S. agalactiae* MCDA assay. Four steps were required for completing *S. agalactiae* MCDA test, including sample collection (3 min), DNA preparation (15 min), MCDA reaction(45 min) and result reporting (6 s)

Two hundreds vaginal and rectal swabs were tested by the MCDA assay, and the results of which were compared to that of enrichment/qPCR and CHROMagar culture. The results showed that *S. agalactiae* infection frequency detected by the MCDA assay was 8.0%, which was in accordance with enrichment/qPCR (Table 3). CHROMagar culture detected 6 samples positive, with the detection rate being 3.0% (Table 3). Each clinical specimen could be completed in 63.1 min from the beginning of the specimen collection until the interpretation of the MCDA assay, which significantly reduced the turn-around time. Thus, the MCDA assay was superior to enrichment/qPCR and CHROMagar culture in detecting *S. agalactiae* directly from vaginal and rectal swabs. The workflow of the *S. agalactiae* MCDA assay was displayed in Fig. 7.

**Table 3:** The detection results of the MCDA assay compared to CHROMagar culture and enrichment/qPCR

MCDA	CHROMagar culture		Enrichment/qPCR	
	Positive	Negative	Positive	Negative
Positive	6	10	16	0
Negative	0	184	0	184

## Discussion

Since the universal screening of *S. agalactiae* was recommended for the women in the late stage of gestation, and the antibiotic was provided to those who tested positive for *S. agalactiae.* As a result, the incidence of neonatal infected with *S. agalactiae* has droped more than 60% (Carrillo-Avila et al. 2018)*.* However, the traditional culture method could not absolutely effective identifying *S. agalactiae.* Then, the rapid and sensitive qPCR assays were introduced to detect *S. agalactiae* in recent years, and several genes frequently have been employed for the specific amplification of *S. agalactiae*, including *cfb* gene and *sip* gene(Cai et al. 2019; Zhao et al. 2019a, b). However, 3.8% of clinical isolates didn’t harbor *cfb* gene and 9.31% of of clinical isolates didn’t have *sip* gene, which may leading false-negative results (Phillips et al. 1980; Elbehiry et al. 2015). Moreover, *cfb* sequences of *S. pyogenes*, *S. uberis* and *S. iniae* have more than 70% sequence similarity to that of *S. agalactiae*, which may lead to false-positive results (Phillips et al. 1980)*.* Thus, *pcsB* gene is alternative to *cfb* gene and *sip* gene for detecting *S. agalactiae.*

MCDA products were usually analyzed by turbidity meter, intercalating dyes, agarose gel electrophoresis and LFB (Wang et al. 2018; Gong et al. 2019; Li et al. 2020). Turbidity meter is an expensive device, which is not easy to obtain in the resource-limited hospitals, and the result is easily suffering from background interference. Although LFB is very portable, the lid of the MCDA reaction tube needed to be opened, increasing the chance of contamination. In this study, intercalating dyes and agarose gel electrophoresis were used to analyze the MCDA products. The results showed that there is no discrepancies on the analysis of the MCDA production between visual MG and 2% gel electrophoresis. However, MCDA products analyzed by 2% gel electrophoresis takes the other 20 min, and the result was obtained by the requirement of the gel imaging system, increasing the run cost. Not like gel electrophoresis, the results obtained by MG was at the end of amplification with naked eyes, eliminating the use of extra equipment and avoiding the risk of aerosol pollution. Thus, visual MG is more practical than 2% gel electrophoresis for analyzing the MCDA products.

This study presents a new isothermal amplification technique on the development of a MCDA diagnostic kit for rapid detection of *S. agalactiae.* The whole experiment only depends on a simple heater, with the weight of 1 kg, which is very easy to achieve in resource-limited restricts, not like an expensive thermal cycler that required for qPCR*.* The detection limit of the MCDA assay was 250 fg, which is more sensitive than 100 pg of PCR and 1 pg of LAMP (Pu et al. 2019)*.* The MCDA assay depends on ten specific primers recognizing 10 different regions of *pcsB* sequence to accomplish amplification. Thus, MCDA in theory is more specific than those nucleic acid amplification methods of using less primer, such as qPCR of two primers and LAMP of six primers. Except for consideration of primers specificity from theoretical design, practical specificity analysis was also evaluated by 28 strains that no cross reactivity occured with non-*S. agalactiae *at 45 min. Thus, the MCDA assay is very portable, and exhibited excellent sensitivity and specificity.

Furthermore, direct detection from vaginal and rectal swabs was examined. It was found that 16 of 200 samples were positive for *S. agalactiae* by MCDA analysis, that being the same as that of enrichment/qPCR assay. This prevelence rate was in the range from 3.7 to 14.52% ever reported (Huang, et al. 2019). Here DNA extraction immediately from clinical samples by heat eliminated the requirement of a complex machine and tedious steps, greatly reducing labor cost and equipment cost. Moreover, the time to complete MCDA detection per clinical specimen was about 63.1 min, which eliminated enrichment culture required for qPCR, saving the time, especially for those at the time of delivery. The cost per test is US$ 4.9, including MCDA per reaction US$ 3.5 and the MG US$ 1.4, which is very economic. All these merits make it possible that the MCDA assay will be a valuable tool for point-of-care diagnosis of *S. agalactiae*.

In conclusion, we have successfully developed a MCDA technique that could immediately detect *S. agalactiae* from vaginal and rectal swabs. This visual method showed excellent sensitivity and specificity. The only device required is a basic heating equipment, which made this method simple for point-of-care test. Further validation with a number of clinical samples was required to evaluate the feasibility of this new developed method.

## Supplementary Information

**Additional file 1: Table S1.** The other two sets of MCDA primers used in this study.

## Data Availability

The raw data supporting the conclusions of this manuscript will be made available by the authors, without undue reservation, to any qualified researcher.
